# Platelet-to-Neutrophil Ratio and Efficacy of Remote Ischemic Conditioning in Acute Ischemic Stroke

**DOI:** 10.1371/journal.pone.0322037

**Published:** 2025-07-03

**Authors:** Yu Cui, Ling-Yun Cui, Xia Chi, Qi Wang, Xian-Wen Zhang, Hui-Sheng Chen

**Affiliations:** 1 Department of Neurology, General Hospital of Northern Theater Command, Shenyang, China,; 2 Department of Neurology, Postgraduate Training Base of Jinzhou Medical University in the General Hospital of Northern Theater Command, Shenyang, China,; 3 Department of Rehabilitation Medicine, General Hospital of Northern Theater Command, Shenyang, China,; 4 Department of Neurology, Mianyang Central Hospital, School of Medicine, University of Electronic Science and Technology of China, Mianyang, China; Chinese Academy of Medical Sciences and Peking Union Medical College, CHINA

## Abstract

**Background:**

Remote ischemic conditioning (RIC) has been proven to improve neurologic function after stroke in RICAMIS trial. Aggravated thrombosis and inflammatory through interaction between platelet and immune cells affected the prognosis of stroke. We conducted an exploratory secondary analysis of RICAMIS to investigate whether platelet-to-neutrophil ratio (PNR) predicted efficacy of RIC in stroke.

**Methods:**

Patients without protocol violation and with baseline blood routine examination from the full analysis set were included and divided into low PNR and high PNR subgroups. The primary outcome was 90-day excellent functional outcome defined as modified Rankin Scale score of 0–1. Compared with usual care alone, we investigated efficacy of RIC treatment in each PNR subgroup and their interaction.

**Results:**

Of 1679 patients, 360 were assigned to low PNR and 1319 into high PNR. Compared with usual care alone, RIC treatment was associated with higher likelihood of 90-day excellent functional outcome across PNR, but significantly different in low PNR (60.9% versus 50.3%, adjusted RD, 11.3%; 95% CI, 1.1% to 21.5%; *P* =0.03) and not significantly different in high PNR (70.8% versus 65.3%, adjusted RD, 3.9%; 95% CI, −1.2% to 8.9%; *P* =0.13). No significant interaction was found (*P* =0.96).

**Conclusions:**

This study firstly investigated the association between PNR at admission and efficacy of RIC treatment in stroke. With respect to long-term functional outcomes, patients benefited from RIC treatment regardless of PNR, but the benefit increased when level of PNR at admission was lower. **Trial registration** ClinicalTrials.gov Identifier: NCT03740971

## Introduction

In the recent published systematic analysis, burden from acute ischemic stroke has increased in the past thirty years, which need effective and accessible therapies to improve stroke surveillance [1]. Accumulating evidence proved that inflammatory responses in the early phase contributed to the poor prognosis of acute ischemic stroke [2–4]. Additionally, platelet activation and aggregation in the acute phase of stroke led to precipitate thrombosis and vascular occlusion [5]. The interaction between platelet and immune cells contributed to aggravation of brain injury after stroke through promoting thrombus formation and inflammation [6,7]. Previous studies investigated the association between several inflammatory ratios such as neutrophil to lymphocyte, platelet to lymphocyte, and lymphocyte to monocyte with neurological recovery following stroke [8,9]. In recent years, platelet to neutrophil ratio (PNR) raised the researchers’ attention as the ratio was associated post-thrombolytic hemorrhagic transformation and long-term outcomes after acute ischemic stroke with higher accuracy [10–13]. Given the closed relationship of inflammatory, thrombosis, and neurological recovery after stroke, it is worth exploring the association between PNR and efficacy of different stroke therapies to identify the potential benefited population.

Remote ischemic conditioning (RIC) was a non-pharmacological therapy blocking the blood flow of limbs and producing transient ischemic with the intention of protecting several organs [14], which also has been demonstrated to improve neurological function in clinical and experimental studies [15,16]. Remote Ischemic Conditioning for Acute Moderate Ischemic Stroke (RICAMIS) trial was the first randomized clinical trial with adequate statistical power to prove that RIC can safely improved 90-day functional outcomes of patients diagnosed with acute moderate ischemic stroke and not receiving reperfusion therapies when RIC treatment was initiated within 48 hours of symptom onset [17]. However, in our recent study, we failed to found the significant association between inflammation and efficacy of RIC treatment based on the data of RICAMIS trial [18]. Considering the relationship between thrombosis and inflammation [6,7], and the effect of RIC based on antithrombosis and anti-inflammation [19], we argued that whether ratios of platelet and immune cells could more affect the efficacy of RIC treatment. Furthermore, as platelet was reported to induce angiogenesis and neurogenesis after cerebral ischemia that was associated with neurological recovery [20,21], PNR may be the target ratio to predict prognosis of patients receiving post-stroke RIC treatment.

Based on above discussion, we explored whether PNR can predict efficacy of RIC treatment after stroke and its suitable level help to distinguish population who benefited more from the RIC treatment.

## Methods

### Study design and participants

Details on the design of RICAMIS have been published [22]. Briefly, RICAMIS trial was a multicenter, randomized clinical trial to assess the efficacy of RIC treatment in patients with acute moderate ischemic stroke. Trial recruited participants from December 26, 2018 to April 19, 2021, and included patient with ≥18 years old, prestroke modified Rankin Scale [mRS] scoring 0–1, and diagnosed with acute moderate ischemic stroke (National Institute Health of Stroke Scale [NIHSS] scores at admission, 6–16) within 48 hours after stroke onset, and excluded those with cardiogenic embolism, contraindication for RIC treatment, or reperfusion therapies. The study was approved by the Ethics Committee of General Hospital of Northern Theater Command (No. k[2018]43) and registered with ClinicalTrials.gov (No. NCT03740971). During the trial, all patients or their legally authorized representatives signed informed consent. Patients from the full analysis set of RICAMIS trial were included in the current study, if they did not violate the trial’s inclusion/exclusion criteria, receive incomplete RIC treatment, or lack the results of blood routine examination at admission.

### Procedures

Blood routine examination collected the counts of platelet, neutrophil, monocyte, and lymphocyte. According to cutoff value of PNR which was proven with better prediction of efficacy of RIC treatment, eligible patients were divided into two subgroups: low PNR and high PNR. The cutoff value of PNR was calculated by the receiver operating characteristics curve in the statistical analysis. In each PNR subgroup, patients were assigned into RIC group and Control group according to whether they received RIC treatment as an adjunct to usual care based on current guideline [23]. RIC treatment was performed by 5 cycles of cuff inflation (200 mm Hg for 5 minutes) and deflation (for 5 minutes), for a total procedure time of 50 minutes, twice daily for 10–14 days [17]. Neurological status, measured with NIHSS score, were evaluated at admission. Follow-up data including NIHSS score and pneumonia during hospitalization, and mRS score and vascular events at 90 days.

### Outcomes

All outcomes in the current study paralleled with those in the RICAMIS [17]. Primary outcome was excellent functional outcome at 90 days (mRS scoring 0–1). Secondary outcomes were favorable functional outcome at 90 days (mRS scoring 0–2), early neurological deterioration at 7 days (an increase between baseline and 7 days of ≥2-points NIHSS score, but not result of cerebral hemorrhage) [24], stroke associated pneumonia at 12 days [25], change in NIHSS score at Day 12, stroke or other vascular events and all-cause death within 90 days. The unblinded assessment of NIHSS score and pneumonia were assessed by investigator at admission. The blinded assessment of mRS score and vascular events within 90 days were conducted by trained investigators in sites who were unaware of assigned treatment.

### Statistical analysis

Primary analyses of the outcomes in the current study were adjusted as the potential imbalance of baseline characteristics between treatment groups after dividing into subgroups according to the level of PNR at admission. For baseline characteristics, we described continuous variables with nonnormal distribution as median (interquartile range [IQR]) and categorical variables as frequencies (percentages). The normal distributions were examined by the Kolmogorov-Smirnov tests and all the continuous variables were nonnormally distributed. The platelet and inflammation associated ratios included platelet to neutrophil ratio, platelet to monocyte ratio (PMR), platelet to lymphocyte ratio (PLR), and systemic immune inflammation index (SII) calculated by multiplying the platelet by the neutrophil and then dividing by the lymphocyte.

First, we respectively calculated the prediction of original model including RIC treatment alone and other four models including each platelet-associated ratio based on the original model for 90-day excellent functional outcome. Binary logistic regression was used to calculate the probability scores of each model, which were then transformed into receiver operating characteristics curve. Unbalanced baseline characteristics between treatments after screening were included in all the models as covariates. The analysis explored PNR as the target ratio with a numerically higher area under the curve and identified the cutoff value of PNR was 30.98. The value of PNR corresponding to shortest distance between the ROC curve and point presenting the 100% true-positive rate and 0% false-positive rate was used as the cutoff value. Furthermore, we explored the association between the probability score and continuous PNR between treatments, and drew the curves with their 95% confidence intervals (CIs). We also evaluated the likelihood of 90-day excellent functional outcome across the PNR level with and without treatments, and calculated the odds ratios (ORs) and 95% CIs.

Second, we compared the efficacy between RIC treatment and usual care alone in PNR subgroups. For binary outcomes such as 90-day excellent functional outcomes, 90-day favorable functional outcome, early neurological deterioration at 7 days, stroke-associated pneumonia at 12 days, and continuous outcomes such as change in NIHSS score at Day 12 compared with baseline, we performed generalized linear models to evaluate the association between treatment and outcomes by generating risk differences (RDs) and 95% CIs. For time-dependent outcomes such as stroke or other vascular events and all-cause death within 90 days, we performed Cox regression analyses to evaluate the association by generating hazards ratios (HRs) and 95% CI. The adjusted analyses will include baseline characteristics with *P* value <0.1 when compared between treatments. Besides the adjusted analyses, unadjusted analyses for all the outcomes were also performed.

Third, the adjusted interaction between PNR subgroups and efficacy of treatments were assessed by generalized linear model or Cox regression model with the treatment groups, PNR subgroups, and their interaction terms as independent variables, imbalance baseline variables between two PNR subgroups with *P* value <0.1 as covariates, and the *P* value presented for the interaction term.

Analyses conducted in the current study were exploratory. Two-sided *P* values less than 0.05 were considered significant. All statistical analyses were completed by SPSS software (Version 26.0, IBM).

## Results

**Fig 1:**
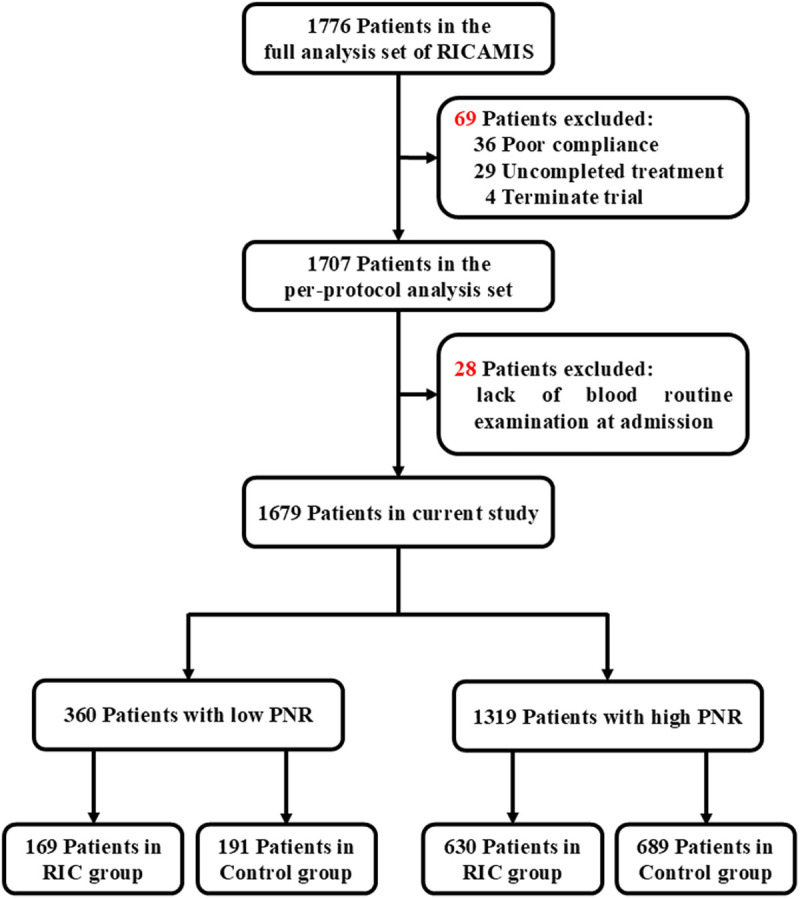
Study Flowchart. Control group included patients who received usual care based on guideline [23] without RIC treatment. Abbreviations: PNR, platelet to neutrophil ratio; RIC, remote ischemic conditioning; RICAMIS, Remote Ischemic Conditioning for Acute Moderate Ischemic Stroke.

**Fig 2:**
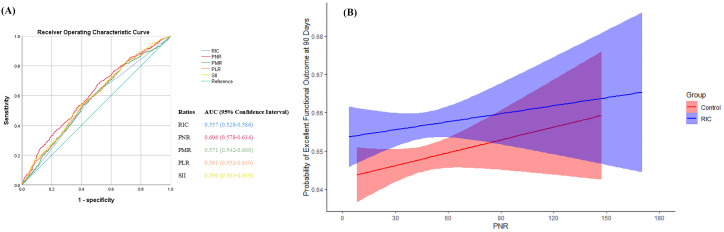
Prediction of PNR for 90-Day Functional Outcome. **(A)** Receiver operating characteristic curve for models to predict 90-day excellent functional outcome. The model of RIC included treatment groups, current drinker, and presumed stroke cause. The models of PNR, PMR, PLR, and SⅡ added these ratios based on the model of RIC, respectively. (B) association between PNR at admission and probability of 90-day Excellent Functional Outcome. The likelihood of 90-day excellent functional outcome was across the PNR with (odds ratio, 1.013; 95% confidence interval, 1.008–1.019; *P* <0.01) and without treatment groups (odds ratio, 1.014; 95% confidence interval, 1.008–1.019; *P* <0.01). Control group included patients who received usual care based on guideline [23] without RIC treatment. Abbreviations: AUC, area under curve; PLR, platelet to lymphocyte ratio; PMR, platelet to monocyte ratio; PNR, platelet to neutrophil ratio; RIC, remote ischemic conditioning; SⅡ, systemic immune inflammation index.

After excluding 69 patients violated the protocol and 28 patients lacked blood routine examination at admission, a total of 1679 patients from the RICAMIS trial were included in the current study (Fig 1). There were some imbalances in the current drinker and presumed stroke etiology between treatment groups among the included patients (Table 1). Compared with RIC treatment, all the four ratios increased the prediction of 90-day excellent functional outcome and PNR showed numerically higher prediction (area under the curve, 0.606; 95% CI, 0.578–0.634), which cutoff value was 30.98 (Fig 2A). According to the cutoff value, patients enrolled in the current study were divided into 360 in the low PNR subgroup and 1319 in the high PNR subgroup. We also found that the probability of 90-day excellent functional outcome increased as the PNR increased in both RIC and Control treatment groups, but the gap of probability between treatment groups increased as the PNR decreased. PNR paralleled with likelihood of 90-day excellent functional outcome with (OR [95% CI], 1.013 [1.008 to 1.019], *P* <0.01) and without treatment groups (OR [95% CI], 1.014 [1.008 to 1.019], *P* <0.01; Fig 2B).

**Table 1:** Baseline Characteristics of Patients Between Treatment Groups.

	RIC (N=799)	Control (N=880)	*P* Value
Age, y	65 (58–72)	66 (58–73)	0.82
Sex (F)	286 (35.8)	287 (32.6)	0.17
Current smoker	230 (28.8)	236 (26.8)	0.37
Current drinker ^a^	119 (14.9)	101 (11.5)	0.04^*^
Comorbidities			
Hypertension	492 (61.6)	532 (60.5)	0.64
Diabetes mellitus	190 (23.8)	211 (24.0)	0.92
Previous ischemic or hemorrhagic stroke ^b^	262 (32.8)	281 (31.9)	0.71
Previous transient ischemic attack	8 (1.0)	10 (1.1)	0.79
Systolic blood pressure, mmHg	150 (140–164)	150 (140–165)	0.30
Diastolic blood pressure, mmHg	90 (80–97)	90 (80–98)	0.21
Blood glucose at randomization, mmol/L	6.50 (5.48–10.23)	6.79 (5.52–10.69)	0.20
NIHSS score at randomization ^c^	7 (6–9)	7 (6–9)	0.75
Onset-to-treatment time, hour	25.7 (14.0–34.7)	25.3 (12.8–35.5)	0.62
Estimated premorbid function (mRS) ^d^			
No symptoms (score, 0)	597 (74.7)	658 (74.8)	0.98
Symptoms without any disability (score, 1)	202 (25.3)	222 (25.2)	
Presumed stroke cause ^e^			
Undetermined cause	458 (57.3)	430 (48.9)	<0.01^*^
Large artery atherosclerosis	208 (26.0)	274 (31.3)	
Small artery occlusion	113 (14.1)	157 (17.8)	
Other determined cause	14 (1.8)	8 (0.9)	
Cardioembolic	6 (0.8)	11 (1.3)	
Platelet Ratios			
Platelet to neutrophil	42.77 (32.77–56.34)	43.56 (32.38–56.71)	0.39
Platelet to monocyte	135.71 (103.94–185.26)	133.24 (103.44–180.30)	0.98
Platelet to lymphocyte	537.04 (400.00–717.86)	535.24 (409.38–711.35)	0.42
Systemic immune inflammation index	641.79 (444.99–1016.82)	637.11 (435.16–1002.55)	0.90

Data were shown with number (percentage,%) or median (interquartile range). mRS, modified Rankin Scale; NIHSS, National Institute of Health Stroke Scale; RIC, remote ischemic conditioning.

^a^Current drinker means consuming alcohol at least once a week within 1 year before onset of the disease and consuming alcohol continuously for more than 1 year.

^b^Previous ischemic stroke referred only to the patients with pre-stroke mRS score ≤1.

^c^Patients with NIHSS scores of 6–16 were eligible for this study; NIHSS scores range from 0 to 42, with higher scores indicating more severe neurologic deficit.

^d^Scores on the mRS of functional disability range from 0 (no symptoms) to 6 (death).

^e^The presumed stroke cause was classified according to the Trial of Org 10172 in Acute Stroke Treatment (TOAST) classification system [29] using clinical findings, brain imaging, and laboratory tests. Other determined causes included pulmonary embolism, peripheral vessel incident, and cardiovascular incident.

* *P* value <0.05.

Between two PNR subgroups (Table 2), there were some imbalances between median systolic blood pressure (153 mmHg in low PNR versus 150 mmHg in high PNR, *P* =0.02), median blood glucose (6.66 mmol/L in low PNR versus 6.16 mmol/L in high PNR, *P* <0.01), and median NIHSS score (8 in low PNR versus 7 in high PNR, *P* <0.01) at randomization. Furthermore, compared with high PNR subgroup, the median platelet count was lower (180×10^9^/L versus 219×10^9^/L, *P* <0.01) and the median neutrophil count was higher (7.56×10^9^/L versus 4.38×10^9^/L, *P* <0.01) in the low PNR subgroup. In the low PNR subgroup, 169 patients received RIC treatment were included in the RIC group, and the remaining 191 patients who received usual care were included in the Control group. Compared with Control group, there were more patients with sex female in the RIC group (37.3% versus 27.2%, *P* =0.04). In the high PNR subgroup, 630 patients who received RIC treatment were included in the RIC group, and the remaining 689 patients who received usual care were included in the Control group. Compared with Control group, there were more imbalances in the current drinker, diastolic blood pressure, and presumed stroke cause in the RIC group. Details of baseline clinical characteristics among groups are shown in Table 3.

**Table 2:** Baseline Characteristics of Patients Between PNR Subgroups.

	Low PNR (N=360)	High PNR (N=1319)	*P* Value
Age, y	67 (60–73)	65 (58–72)	0.12
Sex (F)	115 (31.9)	458 (34.7)	0.32
Current smoker	101 (28.1)	365 (27.7)	0.89
Current drinker ^a^	41 (11.4)	179 (13.6)	0.28
Comorbidities			
Hypertension	227 (63.1)	797 (60.4)	0.36
Diabetes mellitus	83 (23.1)	318 (24.1)	0.68
Previous ischemic or hemorrhagic stroke ^b^	111 (30.8)	432 (32.8)	0.49
Previous transient ischemic attack	5 (1.4)	13 (1.0)	0.51
Systolic blood pressure, mmHg	153 (140–168)	150 (140–164)	0.02^*^
Diastolic blood pressure, mmHg	90 (80–98)	90 (80–98)	0.83
Blood glucose at randomization, mmol/L	6.66 (5.60–9.04)	6.16 (5.33–8.09)	<0.01^*^
NIHSS score at randomization ^c^	8 (6–10)	7 (6–9)	<0.01^*^
Onset-to-treatment time, hour	25.3 (13.4–34.0)	25.5 (13.5–36.0)	0.94
Estimated premorbid function (mRS) ^d^			
No symptoms (score, 0)	277 (76.9)	978 (74.1)	0.28
Symptoms without any disability (score, 1)	83 (23.1)	341 (25.9)	
Presumed stroke cause ^e^			
Undetermined cause	196 (54.4)	692 (52.5)	0.59
Large artery atherosclerosis	106 (29.4)	376 (28.5)	
Small artery occlusion	49 (13.6)	221 (16.8)	
Other determined cause	18 (1.4)	4 (1.1)	
Cardioembolic	5 (1.4)	12 (0.9)	
PNR	25.09 (19.84–28.00)	46.56 (39.61–60.57)	<0.01^*^
Platelet count, ×10^9^/L	180 (147–215)	219 (186–255)	<0.01^*^
Neutrophil count, ×10^9^/L	7.56 (6.20–9.52)	4.38 (3.55–5.40)	<0.01^*^

Data were shown with number (percentage,%) or median (interquartile range). mRS, modified Rankin Scale; NIHSS, National Institute of Health Stroke Scale; PNR, platelet to neutrophil ratio; RIC, remote ischemic conditioning.

^a^Current drinker means consuming alcohol at least once a week within 1 year before onset of the disease and consuming alcohol continuously for more than 1 year.

^b^Previous ischemic stroke referred only to the patients with pre-stroke mRS score ≤1.

^c^Patients with NIHSS scores of 6–16 were eligible for this study; NIHSS scores range from 0 to 42, with higher scores indicating more severe neurologic deficit.

^d^Scores on the mRS of functional disability range from 0 (no symptoms) to 6 (death).

^e^The presumed stroke cause was classified according to the Trial of Org 10172 in Acute Stroke Treatment (TOAST) classification system [29] using clinical findings, brain imaging, and laboratory tests. Other determined causes included pulmonary embolism, peripheral vessel incident, and cardiovascular incident.

* *P* value <0.05.

**Table 3:** Baseline Characteristics of Patients Between Treatment Groups According to PNR.

	Low PNR			High PNR		
	RIC (N=169)	Control (N=191)	*P* Value	RIC (N=630)	Control (N=689)	*P* Value
Age, y	67 (61–73)	67 (59–74)	0.69	65 (58–72)	65 (58–72)	0.86
Sex (F)	63 (37.3)	52 (27.2)	0.04^*^	223 (35.4)	235 (34.1)	0.62
Current smoker	45 (26.6)	56 (29.3)	0.57	185 (29.4)	180 (26.1)	0.19
Current drinker^a^	23 (13.6)	18 (9.4)	0.21	96 (15.2)	83 (12.0)	0.09
Comorbidities						
Hypertension	106 (62.7)	121 (63.4)	0.90	386 (61.3)	411 (59.7)	0.55
Diabetes mellitus	36 (21.3)	47 (24.6)	0.46	154 (24.4)	164 (23.8)	0.79
Previous ischemic or hemorrhagic stroke^b^	46 (27.2)	65 (34.0)	0.16	216 (34.3)	216 (31.3)	0.26
Previous transient ischemic attack	2 (1.2)	3 (1.6)	0.75	6 (1.0)	7 (1.0)	0.91
Systolic blood pressure, mmHg	151 (140–168)	155 (140–169)	0.45	150 (140–162)	150 (140–165)	0.43
Diastolic blood pressure, mmHg	90 (80–98)	90 (80–98)	0.56	90 (80–97)	90 (80–98)	0.08
Blood glucose at randomization, mmol/L	6.47 (5.60–8.70)	6.80 (5.70–9.37)	0.20	6.13 (5.30–7.99)	6.19 (5.36–8.19)	0.77
NIHSS score at randomization^c^	8 (6–11)	8 (6–10)	0.82	7 (6–9)	7 (6–8)	0.58
Onset-to-treatment time, hour	26.0 (13.8–34.1)	24.3 (12.7–34.1)	0.29	25.5 (14.1–35.3)	25.5 (12.8–36.0)	0.99
Estimated premorbid function (mRS)^d^						
No symptoms (score, 0)	131 (77.5)	146 (76.4)	0.81	466 (74.0)	512 (74.3)	0.89
Symptoms without any disability (score, 1)	38 (22.5)	45 (23.6)		164 (26.0)	177 (25.7)	
Presumed stroke cause^e^						
Undetermined cause	98 (58.0)	98 (51.3)	0.58	360 (57.1)	332 (48.2)	<0.01^*^
Large artery atherosclerosis	47 (27.8)	59 (30.9)		161 (25.6)	215 (31.2)	
Small artery occlusion	21 (12.4)	28 (14.7)		92 (14.6)	129 (18.7)	
Other determined cause	2 (1.2)	2 (1.0)		12 (1.9)	6 (0.9)	
Cardioembolic	1 (0.6)	4 (2.1)		5 (0.8)	7 (1.0)	

Data were shown with number (percentage,%) or median (interquartile range). mRS, modified Rankin Scale; NIHSS, National Institute of Health Stroke Scale; PNR, platelet to neutrophil ratio; RIC, remote ischemic conditioning.

^a^Current drinker means consuming alcohol at least once a week within 1 year before onset of the disease and consuming alcohol continuously for more than 1 year.

^b^Previous ischemic stroke referred only to the patients with pre-stroke mRS ≤1.

^c^Patients with NIHSS scores of 6–16 were eligible for this study; NIHSS scores range from 0 to 42, with higher scores indicating more severe neurologic deficit.

^d^Scores on the mRS of functional disability range from 0 (no symptoms) to 6 (death).

^e^The presumed stroke cause was classified according to the Trial of Org 10172 in Acute Stroke Treatment (TOAST) classification system [29] using clinical findings, brain imaging, and laboratory tests. Other determined causes included pulmonary embolism, peripheral vessel incident, and cardiovascular incident.

* *P* value <0.05.

**Fig 3:**
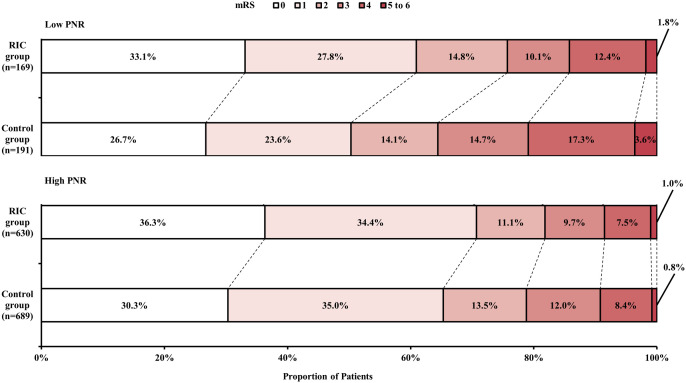
Distribution of 90-Day mRS Score. Scores on the mRS range from 0 to 6. 0 = no symptoms, 1 = symptoms without clinically significant disability, 2 = slight disability, 3 = moderate disability, 4 = moderately severe disability, 5 = severe disability, and 6 = death. Control group included patients who received usual care based on guideline [23] without RIC treatment. Abbreviations: mRS, modified Rankin Scale; PNR, platelet to neutrophil ratio; RIC, remote ischemic conditioning.

We estimated the association between RIC treatment and clinical outcomes in each PNR subgroup. For the 90-day excellent functional outcome, the proportion in the RIC was higher than that in the Control group in the low PNR subgroup with statistical significance (60.9% versus 50.3%; adjusted RD [95% CI], 11.3% [1.1% to 21.5%], *P* =0.03) and in the high PNR subgroup without statistical significance (70.8% versus 65.3%; adjusted RD [95% CI], 3.9% [-1.2% to 8.9%], *P* =0.13). Similar results were also obtained for the 90-day favorable functional outcome, which found statistical significance between treatments in the low PNR subgroup (75.7% versus 64.4%; adjusted RD [95% CI], 11.9% [2.5% to 21.2%], *P* =0.01) and no statistical significance between treatments in the high PNR subgroup (82.1% versus 78.8%; adjusted RD [95% CI], 2.1% [−2.1% to 6.3%], *P* =0.33). The distribution of 90-day mRS score between treatments in each PNR subgroup were shown in Fig 3. For the other outcomes, there was not any significant difference between treatments in any PNR subgroup. For the interaction between PNR and efficacy of RIC treatment, no significant interaction was found. Details of outcome comparisons were shown in Table 4.

**Table 4:** Outcomes Comparison between Treatment Groups according to PNR Subgroups.

Outcomes	PNR	Groups	No. of events (%) or median difference	Treatment effect metric	Unadjusted		Adjusted^a^		*P* Value^b^ for interaction
					Treatment difference (95% CI)	*P* Value	Treatment difference (95% CI)	*P* Value	
mRS scoring 0–1 at 90 days ^c^	Low	RIC (N=169)	103 (60.9)	RD,% ^d^	10.7 (0.5 to 20.9)	0.04	11.3 (1.1 to 21.5)	0.03^*^	0.96
		Control (N=191)	96 (50.3)						
	High	RIC (N=630)	446 (70.8)		5.5 (0.5 to 10.5)	0.03	3.9 (−1.2 to 8.9)	0.13	
			Control (N=689)	450 (65.3)					
mRS scoring 0–2 at 90 days ^c^	Low	RIC (N=169)	128 (75.7)	RD,%^d^	11.3 (2.0 to 20.7)	0.02	11.9 (2.5to 21.2)	0.01^*^	0.41
		Control (N=191)	123 (64.4)						
	High	RIC (N=630)	517 (82.1)		3.3 (−1.0 to 7.5)	0.14	2.1 (−2.1 to 6.3)	0.33	
			Control (N=689)	543 (78.8)					
Early neurological deterioration at 7 days ^e^	Low	RIC (N=169)	18 (10.7)	RD,%^d^	0.7 (−5.6 to 7.0)	0.83	0.4 (−5.8 to 6.6)	0.90	0.61
		Control (N=191)	19 (9.9)						
	High	RIC (N=630)	44 (7.0)		1.3 (−1.3 to 4.0)	0.33	1.5 (−1.1 to 4.1)	0.27	
			Control (N=689)	39 (5.7)					
Stroke−associated pneumonia at 12 days ^f^	Low	RIC (N=169)	10 (5.9)	RD,%^d^	1.7 (−2.8 to 6.3)	0.46	1.8 (−2.8 to 6.3)	0.44	0.97
		Control (N=191)	8 (4.2)						
	High	RIC (N=630)	15 (2.4)		0.8 (−0.7 to 2.3)	0.31	0.7 (−0.8 to 2.2)	0.35	
			Control (N=689)	11 (1.6)					
Change in NIHSS score at day 12 from baseline ^g^	Low	RIC (N=169)	−0.30 (−0.53 to −0.07)	GMR^d^	−0.02 (−0.09 to 0.05)	0.53	−0.03 (−0.09 to 0.04)	0.41	0.60
		Control (N=191)	−0.26 (−0.48 to −0.08)						
	High	RIC (N=630)	−0.35 (−0.66 to −0.15)		−0.03 (−0.06 to 0.01)	0.12	−0.02 (−0.06 to 0.01)	0.23	
			Control (N=689)	−0.30 (−0.54 to −0.10)					
Stroke or other vascular events within 90 days	Low	RIC (N=169)	1 (0.6)	HR^h^	1.13 (0.07 to 18.09)	0.93	0.84 (0.05 to 13.41)	0.90	0.99
		Control (N=191)	1 (0.5)						
	High	RIC (N=630)	4 (0.6)		0.87 (0.24 to 3.26)	0.84	1.04 (0.28 to 3.89)	0.96	
			Control (N=689)	5 (0.7)					
All-cause death within 90 days	Low	RIC (N=169)	2 (1.2)	HR^h^	1.12 (0.16 to 7.98)	0.91	1.16 (0.16 to 8.33)	0.88	0.33
		Control (N=191)	2 (1.0)						
	High	RIC (N=630)	2 (0.3)		0.73 (0.12 to 4.36)	0.73	0.87 (0.14 to 5.26)	0.88	
			Control (N=689)	3 (0.4)					

Abbreviations: CI, confidence intervals; GMR, geometric mean ratio; HR, hazards ratio; mRS, modified Rankin Scale; OR, odds ratio; PNR, platelet to neutrophil ratio; RD, risk difference.

^a^Adjusted for covariates compared between RIC and Control group with *P* value < 0.1 in each age group (sex in the Low PNR; current drinker, diastolic blood pressure, and presumed stroke cause in the High PNR).

^b^Adjusted for covariates compared between the Low PNR and High PNR with *P* value < 0.1 (systolic blood pressure, blood glucose, NIHSS score at randomization).

^c^mRS scores range from 0 to 6: 0 = no symptoms, 1 = symptoms without clinically significant disability, 2 = slight disability, 3 = moderate disability, 4 = moderately severe disability, 5 = severe disability, and 6 = death.

^d^Calculated using generalized linear model.

^e^Early neurological deterioration was defined as an increase between baseline and 7 days of ≥2 on the NIHSS score, but not result of cerebral hemorrhage.

^f^Stroke-associated pneumonia was defined according to the recommendation from the pneumonia in stroke consensus group.

^g^NIHSS scores range from 0 to 42, with higher scores indicating greater stroke severity. Log(NIHSS+1) was analyzed using generalized linear model.

^h^Calculated using Cox regression model. The analyses did not violate the proportional hazard assumption.

* *P* value <0.05.

## Discussion

In this exploratory secondary analysis of the RICAMIS trial, we explored the association between PNR at admission and the efficacy of RIC treatment after acute ischemic stroke. First, We found the consistent tendency between PNR and prognosis of stroke regardless of the treatment, but the difference of treatment effects became larger when PNR was lower. Second, through dividing patients with acute moderate ischemic stroke into low and high PNR subgroups, we found that patients with PNR lower than 30.98 could significantly benefit from the RIC treatment with respect to 90-day functional outcomes, but there was no significant interaction between efficacy of RIC treatment and PNR subgroups.

In the current study, the likelihood of 90-day excellent functional outcome after acute ischemic stroke improved as the baseline PNR increased, which was similar to the results in the previous study [10,11]. This study firstly demonstrated the association efficacy of RIC treatment compared with usual care alone was consistent across the PNR levels. However, the gap of probability of 90-day excellent functional outcome between treatments increased when the PNR was lower at admission. Moreover, we found patients significantly benefited from RIC treatment in lower PNR rather than higher PNR. In the lower PNR subgroup, the counts of platelet was lower and the counts of neutrophil was higher than those in the higher PNR subgroup. As thrombosis induced by inflammation was attributed to interaction between platelets and neutrophil extracellular traps which mainly originated from neutrophil [6], the two counts may indirectly reflect the severer inflammation response at admission. However, in the RICAMIS trial, RIC treatment improved long-term functional outcomes rather than early functional outcomes such as early neurological deterioration and change in NIHSS score at discharge as well as in the current study. Given the inflammation response was associated with neurological deterioration in acute ischemic stroke without reperfusion therpies [26,27], we argued that RIC may contribute to neuroplastic and neurogenesis at later phase rather than anti-inflammation in the early phase [17]. Previous studies have proved that RIC could result in angiogenesis and platelet microparticles induce angiogenesis and neurogenesis after ischemia [20,28]. Thus, based on the above discussion, we interpreted that the efficacy of RIC treatment was significantly better than usual care alone in patients with lower PNR at admission might mainly result from the poor ability of angiogenesis due to lower platelet counts at admission. However, interpretation needs invalidation due to lack of platelet secretion examination and we could not exclude the interaction between RIC treatment and higher neutrophil counts by some potential mechanism.

We admitted there are some limitations in this study. First, about 5.6% of participants were excluded due to several reasons from the full analysis set, which may induce population selection bias. Second, due to the relatively smaller sample size in the low PNR subgroup, this analysis was hampered by inadequate statistical power. Furthermore, the unbalanced sample size between PNR subgroups also limited the interpretation. Third, although the PNR showed numerically higher area under curve, it was not significantly better than the other platelet and inflammation associated ratios. Fourth, the level of PNR with only tested at admission limited the interpretation of the association between PNR and long-term prognosis. Future study with PNR tested in more points will be needed to invalidate the results. Fifth, given the difference of baseline health conditions and genetic predispositions between races, the current findings may not generalize to other populations except China. Finally, the current findings cannot strongly support the PNR as a reliable biomarker in clinical routines. Thus, these findings warrant confirmation.

## Conclusions

In the current study, the efficacy of RIC treatment compared with usual care alone in 90-day excellent functional outcomes kept consistent across the level of PNR at admission, but increased with decreasing PNR in adults with acute ischemic stroke and not eligible for reperfusion therapies. Patients with PNR less than 30.89 at admission may benefit more from RIC treatment. These findings need to be further invalidated.
